# The prevalence of mild cognitive impairment by aspects of social isolation

**DOI:** 10.1371/journal.pone.0269795

**Published:** 2022-06-14

**Authors:** Kyle Masato Ishikawa, James Davis, John J. Chen, Eunjung Lim

**Affiliations:** Department of Quantitative Health Sciences, John A. Burns School of Medicine, University of Hawaii at Manoa, Honolulu, Hawai’i, United States of America; University of Oxford, UNITED KINGDOM

## Abstract

**Objectives:**

This study describes the prevalence of mild cognitive impairment (MCI) across different aspects of social isolation among adults 65 years or older.

**Methods:**

In this cross-sectional study, we utilized the Wave 3 data from the National Social Life, Health, and Aging Project (NSHAP). MCI was defined as a Montreal Cognitive Assessment (MoCA) score less than 23. Prevalence of MCI was calculated for above and below average social disconnectedness (SD), perceived isolation (PI), and demographic variables age, gender, race/ethnicity, education, and household income.

**Results:**

The overall prevalence [and 95% confidence interval] of MCI was 27.5% [25.5–29.6]. The high prevalence of MCI was found in those who had above average SD (32.0% [29.1–34.9]), above average PI (33.3% [29.7–36.8]), were older in age (43.1% [38.9–47.3]), male (28.7% [25.9–31.5]), Black (61.1% [52.5–69.6]), had less than a high school education (66.3% [58.9–73.8]), or were in the lowest income group (46.2% [39.7–52.7]). Those with above average SD or PI had a higher prevalence of MCI in almost all demographics, compared to those with below average SD or PI. Those who were Black or African American or had less than a high school education did not have a greater prevalence of MCI when SD was above average.

**Discussion:**

This current study adds to the body of literature that links SD and PI to MCI and sheds light on the possible existing socio-demographic disparities. Groups with greater than average SD or PI tend to have a higher prevalence of MCI. Further studies are needed to establish a causal association of SD and PI with MCI.

## Introduction

Mild cognitive impairment (MCI) is the transitional stage between normal cognitive functions and early dementia where a person develops memory loss or other cognitive impairment that is not serious enough to interfere with daily activities [1]. The reported prevalence rates of MCI among older adults vary widely in studies across the world from around 3% to 42% [2]. Those with MCI tend to have a lower quality-of-life and greater symptoms of depression and avoid social engagement as a coping mechanism [3]. Sometimes patients with MCI can revert to normal cognitive function, but many times MCI progresses to more serious forms of dementia such as Alzheimer’s disease [4]. The American Academy of Neurology reports that the two year incidence of dementia is 14.9% among patients with MCI aged 65 years or older [5]. Although over 100 drugs have been investigated to slow the progression of dementia or alleviate its symptoms, very few have been authorized for use since 1998 [6]. The Finnish Geriatric Intervention Study to Prevent Cognitive Impairment and Disability (FINGER) was the first study to involve a multi-domain approach to preventing cognitive decline [7]. The study found that older adults who were at-risk of dementia generally improved or maintained their cognitive ability with healthy diet, exercise, cognitive training, and social stimulation.

Social function plays an important role in the mental and physical health of older adults. Cornwell and Waite distinguished two terms, social disconnectedness (SD) and perceived isolation (PI) and described them as the following:

“*Social disconnectedness can be characterized by a lack of contact with others and indicated by situational factors*, *such as a small social network*, *infrequent interaction*, *and a lack of participation in social activities and groups*. *Perceived isolation*, *on the other hand*, *can be characterized by the subjective experience of a shortfall in one’s social resources such as companionship and support* (p. 39) [8].”

Many studies have explored the relationship of PI and/or SD with mental and physical health among older adults. For example, using the National Social Life, Health, and Aging Project (NSHAP) data, Cornwell and Waite found that both SD and PI are independently associated with poorer self-rated physical health but PI has a stronger relationship with mental health than SD [9]. In another study, the influence of SD onto PI is observed with specific mental health illnesses such as depression where PI plays a direct role [10]. A New Zealand study explored the directionality of PI and mental health and found that PI was a better year-to-year predictor of mental health than mental health was to SD [11].

Studies considering both SD and PI often found that both have a significant negative association with cognitive function [12–15]. However, this is not always the case. In a study using data from the China Health and Retirement Longitudinal Study, Yu et al. found that SD, and not PI, was significantly associated with poorer cognitive functions measured at follow-up [16]. Conversely, a study of 2,173 community-living older adults in Amsterdam found that the odds of developing dementia within three years was significantly associated with PI but not SD [17].

To our knowledge, there is a dearth of studies that investigate the association between both SD and PI with MCI among older adults in the United States (US). A study utilizing the NSHAP found that participants with MCI or dementia generally had smaller-sized social networks, less social strain, and less community involvement among US older adults [18]. Although these attributes are part of Cornwell and Waite’s SD measure, the study did not include the aspect of PI. A study conducted in low- and middle-income countries such as China, Ghana, India, Mexico, Russia and South Africa found that PI is significantly associated with MCI in individuals who are 65 years or older but not for those who are younger [19]. This study indicates a significant association between PI and MCI.

In this cross-sectional study, we used the most recent data from the NSHAP (Wave 3) to present the prevalence of MCI among different demographics of an older population and also among those with below and above average SD or PI. Whereas Cornwell and Waite examined two aspects of isolation on self-rated mental health [9], this study is unique because it utilizes a performance-based screening test to assess MCI. As the FINGER study concluded [7], exploring the association between social factors and cognitive decline can improve the psychosocial interventions in the future.

## Methods

### Data source

The NSHAP is a longitudinal population-based study of health and social factors of older Americans with the aim to understand the well-being of older American adults including their physical health, medication use, cognitive function, emotional health, sensory function, health behaviors, social connectedness, sexuality, and relationship quality [20]. So far, the NSHAP has collected three waves of data. In Wave 1 (2005–2006), the NSHAP conducted in-person interviews with 3,005 community-dwelling individuals aged 57–85 years who were born between 1920 and 1947. In Wave 2 (2010–2011), 3,377 interviews were completed with Wave 1 respondents as well as their spouses/partners and those who declined to participate in Wave 1. In Wave 3 (2015–2016), all living participants from Wave 2 and a new cohort of individuals born between 1948 and 1965 were added along with their spouses/partners, totaling 4,777 responses. Detailed information about the survey design and sampling method can be found on the NSHAP website (https://www.norc.org/Research/Projects/Pages/national-social-life-health-and-aging-project.aspx) and the de-identified data is publicly available from the Inter-university Consortium for Political and Social Research (ICPSR) repository (https://www.icpsr.umich.edu/web/NACDA/studies/36873). This study was deemed exempt from review by University of Hawaiʻi Institutional Review Board (IRB #2021–00780).

### Measures

The Montreal Cognitive Assessment (MoCA) is a validated 30-point screening tool for MCI [21]. This assessment covers eight domains of cognition including short-term memory, visuospatial abilities, executive function, attention, concentration, working memory, language, and orientation to space and time. An individual’s MoCA score was the total points earned from answering the assessment questions correctly. The reliability of this measure was acceptable in this study (Cronbach’s alpha = 0.75). In this study, any participant who scored below a cutoff threshold of 23 were considered at higher risk of MCI [22–24].

Each participant’s SD score was calculated using Cornwell and Waite’s method [8]. SD incorporates social network size and range, frequency of interaction with network members, proportion of network members in the home, number of friends, attendance at group meetings, socializing with friends and family, and volunteering. Each variable was recoded such that greater points indicated connectedness. Next, the responses for each question were standardized before the responses for each participant were averaged and reversed to create the SD scores. If a participant could answer at least one question regarding SD, their respective SD score could be calculated. The SD scores ranged from -1.19 to 1.93 and a SD score above zero indicated greater than the average SD. We computed a Cronbach’s alpha of 0.66 for SD in this study and determined the reliability was acceptable according to Hulin et al. [25].

The score of PI was also calculated using Cornwell and Waite’s method [8]. PI is comprised of emotional and instrumental support from family members, friends, and spouse or partner; lack of companionship; feeling left out; and feeling isolated. The latter three items are questions from the University of California at Los Angeles three-item loneliness scale [26]. Each response was recoded such that greater points indicated loneliness. Next, the responses for each question were standardized before the responses for each participant were averaged to create the PI scores. These scores ranged from -1.06 to 3.34 and a PI score above zero indicated greater than average PI. The reliability of PI in this study was acceptable (Cronbach’s alpha = 0.68).

The following socio-demographic variables were considered as covariates–age, sex, education, race/ethnicity, and income. These variables were known to be associated with MCI among older adults [12, 27]. Education was categorized into four groups: less than high school, high school or equivalent, some college including vocational certificate and associate, and bachelors or more. Race was categorized as White, Black, and Other which included Asian, Pacific Islander, American Indian or Alaskan Native. Lastly, household income was categorized as $0-$24,999; $25,000-$49,999; $50,000-$99,999; and $100k or higher.

### Study sample

**Fig 1:**
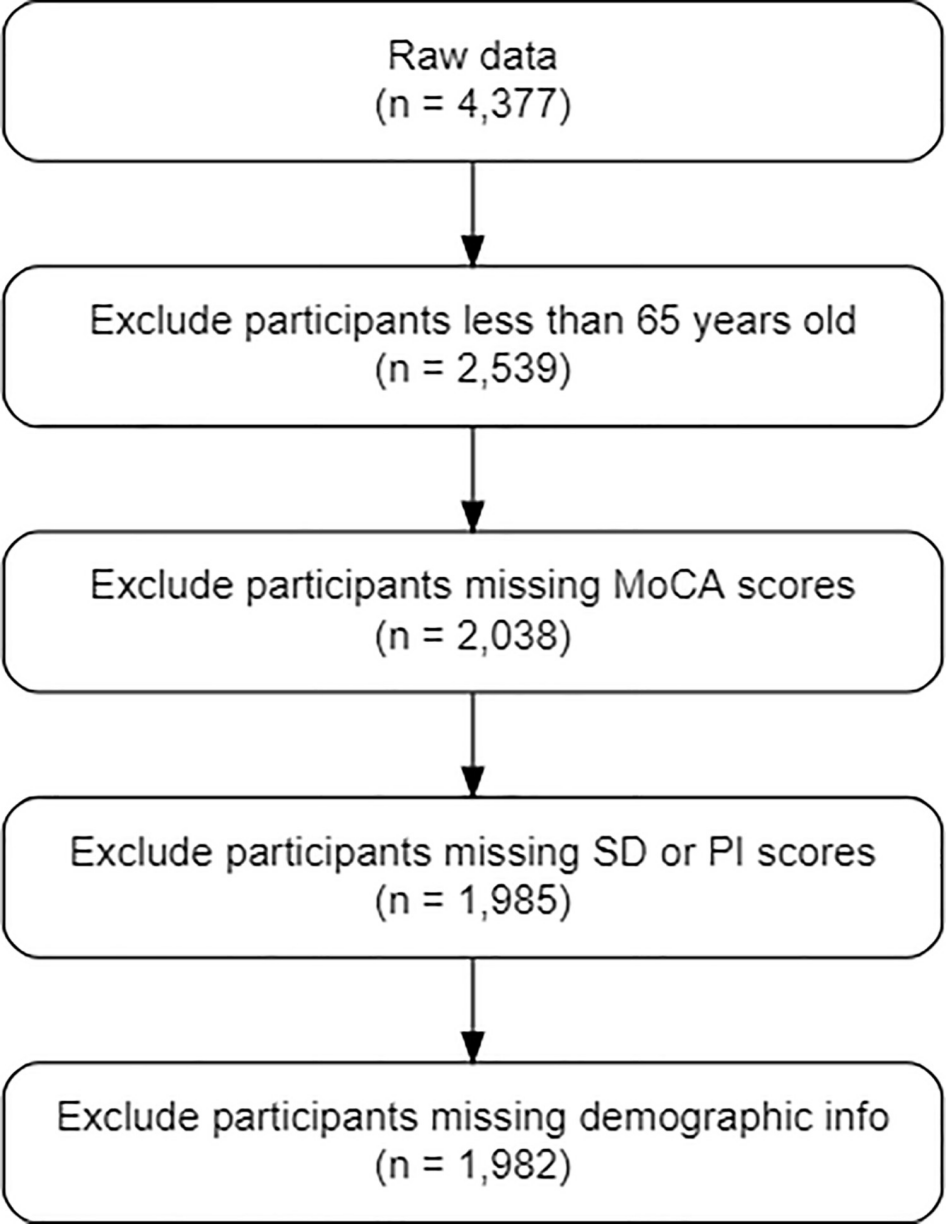
Study flow chart.

This study is cross-sectional and only used Wave 3 of the NSHAP because it had the largest amount of MoCA scores available. The inclusion criteria for this study were adults 65 years of age or older with MoCA, SD, and PI scores. Fig 1 depicts the flowchart of our study sample. The final data include 1,985 older adults.

### Statistical analysis

Descriptive statistics were reported using unweighted frequencies and weighted proportions for SD, PI, and socio-demographic variables. SD, PI, and age were dichotomized to report frequencies, but were analyzed in continuous forms as well. Lastly, weighted percentages of MCI for above and below average SD and PI were calculated for each demographic. All analyses were implemented in R version 4.0.3 and the survey package was used to adjust for the complex sampling design of the NSHAP.

## Results

A total of 1,985 participants aged 65 to 95 years with complete scores for MoCA, SD, and PI scores were included in the final analysis. Of these participants, 655 (27.5% weighted) were considered at higher risk for MCI (Table 1). Greater weighted proportions of MCI were seen in those who had above average SD or PI; were older in age, male, less educated, or Black; or had lower household income. Table 2 presents weighted mean and standard error for continuous variables. The weighted mean for SD, PI, and age of those with MCI tended to be higher than those without MCI. Participants who were 65 years or older and excluded from the study due to missing MoCA or PI scores (S1 Table) had a greater proportion of above average SD, were female, older, Black or the Other race, or had the lowest educational attainment or household income. No one older than 65 years had missing SD scores.

**Table 1:** Prevalence of mild cognitive impairment by socio-demographics.

Variable	Unweighted N	Weighted % [95% CI]	Unweighted N with MCI	Weighted % [95% CI] with MCI
**Total**	1,985	100	655	27.5 [25.5–29.6]
**Social Disconnectedness**				
Average or below	1,072	54.1 [51.2–57.0]	291	23.7 [20.7–26.8]
Above average	913	45.9 [43.0–48.8]	364	32.0 [29.1–34.9]
**Perceived Isolation**				
Average or below	1,139	58.7 [56.2–61.2]	332	23.5 [20.5–26.4]
Above average	846	41.3 [38.8–43.8]	323	33.3 [29.7–36.8]
**Gender**				
Female	1,045	52.3 [49.8–54.8]	320	26.4 [23.3–29.6]
Male	940	47.7 [45.2–50.2]	335	28.7 [25.9–31.5]
**Age**				
65–74 years old	1,190	68.7 [66.3–71.0]	296	20.4 [18.0–22.8]
75 years and older	795	31.3 [29.0–33.7]	359	43.1 [38.9–47.3]
**Education**				
< High school	200	8.6 [6.9–10.2]	147	66.3 [58.9–73.8]
High school or equivalent	481	24.5 [21.6–27.4]	176	30.4 [25.2–35.6]
Vocational certificate, some college, or associate	646	34.4 [31.5–37.3]	226	29.3 [25–33.5]
Bachelors or more	618	32.6 [28.7–36.4]	106	13.2 [10.4–16.1]
**Race**				
White/Caucasian	1,649	87.9 [85.8–90.0]	446	24.1 [21.9–26.3]
Black/African American	217	7.3 [5.6–9.0]	137	61.1 [52.5–69.6]
Other (Asian, Pacific Islander, American Indian or Alaskan Native)	116	4.8 [3.4–6.3]	51	39.0 [27.8–50.3]
**Income**				
$0-$24,999	311	20.2 [16.9–23.4]	167	46.2 [39.7–52.7]
$25,000-$49,999	453	26.5 [23.1–29.7]	155	29.0 [24.9–33.1]
$50,000-$99,999	565	35.6 [32.5–38.6]	128	18.1 [14.4–21.9]
$100,000 or higher	269	17.8 [14.8–20.9]	42	12.6 [8.2–17.0]

MCI = Mild Cognitive Impairment. CI = Confidence Interval.

**Table 2:** Weighted mean of continuous variables by mild cognitive impairment status.

Variable	Mean (SE) without MCI	Mean (SE) with MCI
Social Disconnectedness	-0.052 (0.014)	0.060 (0.022)
Perceived Isolation	-0.075 (0.019)	0.103 (0.030)
Age (years)	72.0 (0.2)	76.0 (0.3)

SE = Standard Error. MCI = Mild Cognitive Impairment.

Lastly, when comparing those with average SD or PI to those with below average SD or PI (Table 3), the above average group almost always tended to have a higher proportion of MCI. The exceptions to that pattern were those with less than a high school education or Blacks or African Americans. These groups had slightly smaller proportions of MCI when their SD was above average.

**Table 3:** Weighted proportion [and 95% confidence interval] of mild cognitive impairment by social disconnectedness and perceived isolation.

	Social Disconnectedness		Perceived Isolation	
Variable	Average or Below	Above Average	Average or Below	Above Average
**Gender**				
Female	21.7 [18.0–25.5]	32.8 [28.1–37.6]	22.3 [18.3–26.3]	33.3 [28.3–38.3]
Male	26.3 [21.5–31.0]	31.2 [26.2–36.2]	24.9 [20.7–29.1]	33.3 [27.8–38.7]
**Age**				
65–74 years old	18.5 [15.1–21.9]	22.8 [19.0–26.6]	16.3 [13.2–19.3]	26.6 [21.9–31.4]
75 years and older	36.5 [29.8–43.3]	49.7 [43.9–55.4]	40.6 [34.3–46.8]	46.3 [41.2–51.4]
**Education**				
< High school	67.0 [51.6–82.5]	65.9 [54.3–77.5]	60.7 [48.6–72.7]	71.1 [60.0–82.2]
High school or equivalent	25.9 [19.5–32.4]	35.1 [28.4–41.8]	27.3 [19.9–34.8]	34.4 [27.1–41.8]
Vocational certificate, some college, or associate	26.3 [21.1–31.5]	32.8 [26.3–39.3]	25.9 [19.5–32.3]	34.6 [28.0–41.2]
Bachelors or more	12.5 [7.5–17.5]	14.3 [10.3–18.3]	10.8 [7.9–13.6]	17.1 [10.6–23.5]
**Race**				
White/Caucasian	20.3 [17.2–23.3]	28.8 [25.5–32.1]	20.9 [17.9–23.9]	28.8 [25.1–32.6]
Black/African American	62.8 [50.3–75.2]	59.2 [48.2–70.2]	54.9 [43.7–66.1]	67.9 [54.7–81.0]
Other (Asian, Pacific Islander, American Indian or Alaskan Native)	33.4 [14.5–52.4]	43.3 [26.9–59.7]	29.4 [13.6–45.2]	49.0 [32.3–65.6]
**Income**				
$0-$24,999	44.4 [35.0–53.9]	47.7 [39.6–55.7]	43.8 [36.0–51.7]	48.9 [39.6–58.3]
$25,000-$49,999	25.6 [19.2–31.9]	32.4 [26.0–38.8]	26.7 [21.0–32.3]	32.0 [25.3–38.7]
$50,000-$99,999	16.2 [10.9–21.4]	21.1 [15.4–26.9]	13.8 [9.7–17.9]	26.3 [18.5–34.1]
$100,000 or higher	10.9 [5.5–16.3]	15.2 [7.3–23.1]	8.9 [4.6–13.2]	18.6 [9.1–28.1]

## Discussion

Using the nationally representative NSHAP data, we estimated the prevalence of MCI among socioeconomic demographics of older adults in US. We found that the prevalence of MCI among the older population was 27.5%. The prevalence rate of MCI in our study is comparable to existing research. For example, in a systematic review of 35 studies, Ward et al. reported a median prevalence of 26.4% but noted that the range of prevalence was broad within and across different classifications of MCI [2]. Our study adds to the body of literature that discusses the prevalence within older demographic groups and between different levels of social isolation.

Loneliness has numerous dimensions including *intimate*, *relational*, and *collective* loneliness [28]. Intimate loneliness is the feeling of lacking a social network based on emotional support; relational loneliness is based on perceived connections with others; and collective loneliness is based on an individual’s social network and identities that offer low-cost, impersonal support. The PI score used in this study represents intimate loneliness, while SD represents relational and collective loneliness.

Social isolation and MCI are health risk factors which need to be addressed. Using technology to maintain intimacy can work in the case of text messaging with emoticons [29], but there is limited evidence that video calling reduces SD and PI [30]. Teaching older adults how to use video calling may not address a deeper underlying cause of PI, such as lack of family commitment [31]. According to a meta-analysis of interventions to reduce PI, the most successful intervention is treating maladaptive social cognition [32]. People who are aware of their own cognitive decline may avoid social activities as a coping mechanism [3], making it hard to alleviate loneliness. This observation supports a regulatory model that includes PI, insecurity, hypervigilance, negative social expectation, defensive emotions, and distancing; each cascading to one another and perpetuating the loop [33]. Older adults are more likely to enter the loop because, in addition to their declining cognition, they are more likely to experience one or more events that worsen loneliness such as the loss of a loved one, chronic health problems, sensory impairment, and income instability [34]. In another study which used the MoCA to determine MCI status, those with MCI had a significantly lower quality of life and negative affective reactivity, which is the response to cognitive difficulty [35]. In a study of 823 old adults without dementia in Chicago, loneliness was associated with lower cognition at baseline and quicker cognitive decline during follow-up [36]. A meta-analysis also found that loneliness was significantly associated with increased risk for dementia, although it did not have enough evidence to support the association between loneliness and MCI [37]. Therefore, it is important to identify feelings such as PI, insecurity, hypervigilance, and negative social expectation that are associated with loneliness and then determine their longitudinal relationship with cognitive impairment.

Higher prevalence of MCI was observed in participants who were 75 years or older, were Black or of the Other race group, had less education, or had less household income. Studies have shown that MoCA performance varies significantly by age and educational attainment [38–40]. Age has a negative correlation with MoCA score while higher levels of education have a positive correlation. Accordingly, older age and less education are associated with higher frequencies of MCI [41]. In our results, we can see a steady decrease in proportions of MCI as education or income increases. Blacks in particular had lower MoCA scores which is consistent with past studies [42, 43]. This could not be explained by educational attainment or household income stratified by race because the Other race group had the highest proportion of individuals who had less than a high school education or made less than $25,000 per year. Further investigation is needed to explain the disparity of MCI among the Black population.

Our results should be interpreted considering the following limitations. First, although the NSHAP is a longitudinal study, we used only Wave 3 data due to the completeness of responses to MoCA questions. Thus, we did not explore the temporal change of MCI or causation of SD or PI on MCI. Investigating which factors are associated with the conversion from normal cognition to MCI is crucial because healthcare providers can gain insight for the development of an early intervention that can prevent or delay the onset of MCI or dementia. This issue may be studied when the Wave 4 data is released. Second, we used the cutoff point of 23 to identify MCI. As mentioned above, some studies suggest using different cutoffs stratified by race/ethnicity and education to ensure detecting MCI from non-MCI from dementia. Using a different cutoff point could lead to different results. However, further research is needed to investigate and validate MoCA cutoff points. Third, the exclusion of participants with incomplete responses to the MoCA could lead to bias because those who did not answer all assessment questions could have gotten lower MoCA scores than those included in the study. As such, those who were excluded from the study had the same characteristics as those with the highest prevalence of MCI.

Despite these limitations, this study has several strengths. First, the NSHAP selected participants using a national frame from either the Health and Retirement Study (participants from Wave 1) or National Opinion Research Center (NORC)’s national frame (additional participants added in Wave 3) [20]. This study incorporates the sampling design to produce weighted results which are likely generalizable to the US older adult population. Second, unlike other studies that used self-rated mental health, this study utilized a performance-based test, the MoCA.

In conclusion, using a nationally representative large sample, we found that across almost all demographics, those with greater than average SD or PI have greater proportions of MCI than those with average or below average SD or PI, respectively. This current study adds to the body of literature that describes the prevalence of MCI among different aspects of social isolation.

## Supporting information

S1 Table Characteristics of those excluded from the study. (DOCX)
