# Supplementary material for: The prevalence of mild cognitive impairment by aspects of social isolation
Source: PLoS One. 2022 Jun 14;17(6):e0269795. doi: 10.1371/journal.pone.0269795 (PMC9197049; doi:10.1371/journal.pone.0269795)
Supplement: S1 Table — (DOCX) [file pone.0269795.s001.docx]

**Table 1. Characteristics of Those Excluded from the Study**

| **Variable** | **Unweighted N** | **Weighted %**  **[95% CI]** |
| --- | --- | --- |
| **Total** | 554 | 100 |
| **MCI Status** |  |  |
| No MCI | 24 | 49.0 [25.3-72.7] |
| MCI | 29 | 51.0 [27.3-74.7) |
| **Social Disconnectedness** |  |  |
| Average or below | 206 | 38.7 [33.4-44.0] |
| Above average | 348 | 61.3 [56.0-66.6] |
| **Perceived Isolation** |  |  |
| Average or below | 259 | 60.2 [54.6-65.9] |
| Above average | 199 | 39.8 [34.1-45.4] |
| **Gender** |  |  |
| Female | 340 | 62.9 [58.3-67.5] |
| Male | 214 | 37.1 [32.5-41.7] |
| **Age** |  |  |
| 65-74 years old | 228 | 53.4 [48.1-58.7] |
| 75 years and older | 326 | 46.6 [41.3-51.9] |
| **Education** |  |  |
| < High school | 221 | 31.5 [23.6-39.5] |
| High school or equivalent | 145 | 28.6 [23.1-34.2] |
| Vocational certificate, some college, or associate | 118 | 24.9 [19.6-30.3] |
| Bachelors or more | 70 | 14.9 [10.6-19.2] |
| **Race** |  |  |
| White/Caucasian | 332 | 73.4 [67.6-79.2] |
| Black/African American | 141 | 15.9 [11.5-20.2] |
| Other (Asian, Pacific Islander, American Indian or Alaskan Native) | 76 | 10.7 [6.1-15.4] |
| **Income** |  |  |
| $0-$24,999 | 173 | 45.8 [37.9-53.8] |
| $25,000-$49,999 | 94 | 24.9 [19.3-30.5] |
| $50,000-$99,999 | 56 | 20.5 [14.2-26.8] |
| $100,000 or higher | 25 | 8.8 [4.9-12.6] |

MCI = Mild Cognitive Impairment. CI = Confidence Interval
